# Optimising Radiation Dose Estimation: UNSCEAR DAP‐to‐ED Conversion in Uterine Artery Embolisation

**DOI:** 10.1002/jmrs.70002

**Published:** 2025-06-19

**Authors:** Don J. Nocum, John Robinson, Warren Reed

**Affiliations:** ^1^ Discipline of Medical Imaging Science Faculty of Medicine and Health, Sydney School of Health Sciences, the University of Sydney Sydney New South Wales Australia; ^2^ Ramsay Health Care, North Shore Private Hospital St Leonards New South Wales Australia

**Keywords:** angiography, dose, effective dose, radiation effects, vascular interventional

## Abstract

**Introduction:**

Radiographers and physicians working in interventional radiology (IR) departments are responsible for monitoring and optimising radiation dose exposure to both patients and staff. The dose‐area product (DAP) is a common measurement of radiation output but does not directly correlate with stochastic risks. Pre‐determined conversion factors allow estimation of effective dose (ED) for IR procedures to better assess radiation exposure risks. This study evaluates the clinical utility of DAP‐to‐ED conversion factors to improve knowledge of radiation risk assessment.

**Methods:**

Retrospective data on DAP (Gray per centimetre‐squared/Gy.cm^2^) from uterine artery embolisation (UAE) procedures were analysed. Conversion factors were obtained from the ‘United Nations Scientific Committee on the Effects of Atomic Radiation (UNSCEAR) Global Survey on Medical Exposure: A User Manual’. Group A (*n* = 50), which followed standard protocols, was compared with Group B (*n* = 50) which implemented dose optimisation techniques.

**Results:**

Multivariable linear regression (MVLR) analysis demonstrated that DAP correlated with the converted ED values for both groups (*p* < 0.01). The mean ED was 9.5 milliSieverts (mSv) for Group A and 8.7 mSv for Group B.

**Conclusion:**

MVLR analysis confirmed a strong correlation between DAP and the ED conversions, demonstrating that the ‘UNSCEAR User Manual’ has potential to serve as a DAP‐to‐ED estimation tool for common interventional procedures. The mean ED found was equivalent to the radiation dose of approximately one abdominal computed tomography (CT) scan. Implementing DAP‐to‐ED conversion can be valuable in improving both clinicians and patients' awareness of radiation exposure.

## Graphical abstract

This study uses the conversion factors from the ‘UNSCEAR Global Survey on Medical Exposure: A User Manual’ to estimate radiation dose exposure for patients undergoing uterine artery embolisation. Estimating the effective dose provides interventional radiologists and interventional radiographers a better understanding of the stochastic risks associated with a specific interventional radiology procedure and the importance of dose optimisation practices.

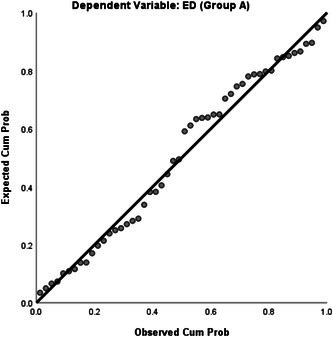

## 1 Introduction

The measurement of patient radiation dose exposure during diagnostic and interventional procedures is critical for ensuring patient safety and implementing the appropriate radiation protection protocols [1]. The dose‐area product (DAP) is commonly used to quantify the total radiation delivered to the patient, expressed as a product of emitted radiation dose and the area irradiated [2, 3]. While DAP effectively measures radiation dose output from the angiography unit, both during and after the procedure, it does not consider the varying sensitivities of different tissues to radiation exposure [4]. Effective dose (ED) provides a more comprehensive assessment of radiation exposure in patients by integrating radiation type and organs sensitivity, making it a preferred metric for evaluating overall stochastic risk to the patient [5]. In clinical practice, ED is not routinely calculated by angiography units or operators. Instead, DAP and air kerma (AK) remain the primary metrics for assessing stochastic and deterministic risks, respectively [6, 7]. Determining ED, measured in millisieverts (mSv) or sieverts (Sv), offers a more appropriate metric for assessing radiation exposure in angiography, where radiation levels normally exceed those used in general X‐ray or computed tomography (CT) [8].

The interventional radiology (IR) procedure in focus in this study is uterine artery embolisation (UAE), which involves the embolisation of fibroids and/or adenomyosis in pre‐menopausal women, manifesting as benign growths in the uterus and symptomatic with menorrhagia and dysmenorrhoea [9, 10]. Ionising radiation is used for angiographic imaging and guidance of the treatment process through embolisation of the uterine arteries [11, 12]. Since the patient population undergoing UAE is of reproductive age and recent studies have shown that pregnancy is viable post‐UAE [13], it is important to optimise radiation dose exposure during this procedure. Understanding the radiation dose beyond DAP and AK values and determining ED measurements are indicative of any potential stochastic risks when radiating sensitive reproductive organs.

Radiological protection is based on the three principles of justification, optimisation, and responsibility for radiation dose reduction [4]. Interventional radiologists and radiographers follow various guidelines depending on their countries regulations and available resources. The International Atomic Energy Agency (IAEA) provides recommendations for optimising radiation dose practices during diagnostic and interventional procedures, summarising common mean ED values from the literature. These include endovascular aortic repair (EVAR) (ED = 8.7–27 mSv) [14, 15], venous access procedures (ED = 1.2 mSv) [16], renal/visceral angioplasty ± stent (ED = 54 mSv) [3], and iliac angioplasty ± stent (ED = 58 mSv) [3]. Some institutions rely on National Diagnostic Reference Levels (NDRLs) to guide practice, such as the United Kingdom's (UK) NDRLs for general radiography and fluoroscopy, established in 2016 [17]. However, these NDRLs only consider DAP and the fluoroscopy time per procedure [17]. In Australia, the Australian Radiation Protection and Nuclear Safety Agency (ARPANSA) has only developed NDRLs for coronary angiograms and is currently surveying sites for image‐guided interventional procedures and relevant dosimetry data [18]. In 2017, the United Nations Scientific Committee on the Effects of Atomic Radiation (UNSCEAR) developed the ‘UNSCEAR Global Survey for Medical Exposure: A User Manual’ (abbreviated to ‘UNSCEAR User Manual’) [19] which provides dose conversion coefficients (DCCs) for estimating ED from DAP values for common diagnostic and IR procedures. This study utilises the ‘*UNSCEAR User Manual’* to evaluate the feasibility of applying DAP‐to‐ED conversions in a clinical setting [14].

The ‘UNSCEAR User Manual’ provides the dose conversion coefficient (DCCE), measured in [mSv.(Gy.cm^2^)^−1^], for a range of interventional and vascular procedures [19]. By multiplying the DAP (Gy.cm^2^) from an angiography unit with the DCCE, the relative ED for a specific patient and procedure can be determined [19]. This approach is practical and accessible for clinicians, without extensive research or use of proprietary software programmes such as the Personal Computer Program for X‐ray Monte Carlo (PCXMC v2.0, Vataa, Finland) [20, 21]. Kriechenbauer et al. [22] used the same DCCE of 0.26 mSv.(Gy.cm^2^)^−1^ for prostate artery embolisation procedures to determine the ED for males treated for benign prostatic hyperplasia, where the ED for Group I was 146.6 and 64.2 mSv for Group II. This study considers that the DAP‐to‐ED conversions are not absolute and are to be used to provide interventional radiographers and radiologists an estimate indication of ED for patients, particularly when the department does not have a resident medical physicist on‐site. Despite the availability of conversion coefficients in the ‘UNSCEAR User Manual’, the routine use of ED calculations in clinical practice in the literature remains limited. Huo and colleagues [23] used anthropometric patient phantoms to conduct Monte Carlo simulations and calculate organ doses in IR, demonstrating that organ dose values (mSv) increase proportionally with tube voltages and decrease as the absorber thickness increases, due to the shielding of low energy X‐ray and reduced X‐ray intensity [23]. Similarly, Two studies [24, 25] independently used PCXMC v2.0 (Vataa, Finland) to determine the ED for paediatric patients undergoing three‐dimensional rotational angiography (3DRA) for cardiac investigations. Both studies demonstrated correlations between DAP and ED, enabling the optimisation of radiation dose exposure for their patient cohorts [24, 25]. Another study [26] developed local diagnostic reference levels (LDRLs) for angiographic and fluoroscopic procedures based on 11,000 examinations over a 2.5‐year period. Their work informed Australian practice on radiation dose using DAP values, addressing the absence of an NDRL and a widely accessible method for calculating ED [26]. This study aims to evaluate the use of the ‘UNSCEAR User Manual’ and integrate appropriate ED calculations into clinical practice across various IR and angiography suites.

Therefore, the purpose of this study was to determine the clinical use of conversion factors for DAP to ED and improve knowledge about radiation and its associated risks. The conversion process should be accessible and reliable, allowing interventional radiographers and radiologists to perform estimated DAP‐to‐ED calculations in clinical settings to determine an indication of ED. Evaluating the conversion coefficients provided by UNSCEAR can support the optimisation of radiation dose practices and enhance understanding of radiation dosimetry and radiation risks for IR procedures where higher radiation dose exposures may be involved.

## 2 Methods

### 2.1 Study Setting

This study was exempted from ethical review by the Adventist HealthCare Limited Human Research Ethics Committee (HREC 2018‐004) as it met the requirements from the National Statement on Ethical Conduct in Human Research 2023; Sections 5.1.17(a). Retrospective case–control data were collected from 100 patients who had undergone a UAE procedure using a Philips Azurion with FlexArm (Philips Healthcare, Best, the Netherlands) angiography system. The exposure data were used to convert the DAP to ED. Group A consisted of 50 patients (age range 34–54 years old) for whom digital subtraction angiography (DSA) was used to visualise the abdominal aortogram, left and right internal iliac arteries, and left and right uterine arteries. Group B included 50 patients (age range 29–53 years old) for whom DSA was used to image the abdominal aortogram and left and right uterine arteries, while a conventional roadmap (CRM) technique was employed to image the left and right internal iliac arteries. Live fluoroscopy was used to guide the passage of image guidewires and catheters/microcatheters and to monitor the embolisation phase using particulate embolics (Contour Polyvinyl Alcohol (PVA), Boston Scientific, United States). The use of CRM in Group B was intended to measure the differences in the reduction of radiation dose compared to DSA, while still providing sufficient image quality for internal iliac artery visualisation and without detriment to fluoroscopy time.

### 2.2 Angiography Unit

The angiography system used pulsed fluoroscopy at 7.5–15 pulses per second (pps) and DSA at 2 frames per second (fps) for 3 s, followed by 1 fps for 2 s, and finally 0.5 fps for the duration of the exposure. Added filtration of 1.0 mm Aluminium (Al) and 0.1 mm Copper (Cu) was applied. The kilovolt (kVp) accuracy and total tube filtration were measured annually as part of a quality assurance programme. Digital magnification from the 48 cm flat‐panel detector (FD) and collimation were applied during the procedures based on the clinical and radiological requirements of a single interventional radiologist.

The DAP was measured using a calibrated DAP meter (Kerma X‐plus; Ion Beam Applications (IBA) Dosimetry, Schwarzenbruck, Germany) mounted on the exit surface of the collimator assembly. The DAP meter was calibrated using the Unfors Xi dosimeter system (Raysafe, Billdal, Sweden) and the User Quality Control Mode software (Philips Healthcare, Best, Netherlands). The AK readings were measured in Gy and the detector field size (cm^2^) was recorded as the fraction of the DAP over AK.

### 2.3 Statistical Analysis

International Business Machines (IBM) SPSS Statistics v29.01.0 (Armonk, New York, USA) was used to perform descriptive statistics and paired sample *t*‐tests for the demographic and clinical information of patients in Group A and Group B, as well as their radiation dosimetry and conversion values. A multi‐variable linear regression (MVLR) analysis was conducted separately for Group A and Group B patients to evaluate the correlation between ED and the study variables.

### 2.4 DAP to ED Conversion

The ‘UNSCEAR User Manual’ was used to convert the DAP values to ED, referencing ‘Factors for estimation of effective dose from image‐guided interventional procedures’ (page 35 of 64). For ‘non‐cardiac interventional vascular procedures’, a DCCE of 0.26 [mSv.(Gy.cm^2^)^−1^] (specific to pelvic arterial embolisation) was used to calculate ED (mSv) from the DAP values [19]. If the DAP units on a particular angiography unit are not expressed in Gy.cm^2^, a DAP unit converter was used for an accurate ED calculation [27]. An additional analysis of the ED calculation was performed using the ED correction factors as described by Huo and colleagues [23] to correct for the variations in patient body mass index (BMI).

## 3 Results

The complete summary of patient demographic and clinical information for Group A (*n* = 50) and Group B (*n* = 50) participants are reported in Table 1. The paired samples *t*‐test results indicate that BMI was statistically significant (*p* < 0.01).

**TABLE 1:** Group A and Group B patient demographic and clinical information.

Variable	Group A, mean ± SD, (median) [range]	Group B, mean ± SD, (median) [range]	Paired samples *t*‐test, *p*, Cohen's *d*
Age (years)	44 ± 5 (43) [34–54]	44 ± 5 (45) [29–53]	0.866, −0.06
BMI (kg/m^2^)	24.7 ± 4 (24.1) [16.5–33]	24.3 ± 3 (24.4) [19.1–33.4]	< 0.001, 0.25^a^
Total uterus volume (cm^3^)	292 ± 246 (184) [47–995]	471 ± 286 (352) [72–1550]	0.153, −0.43

Abbreviations: BMI, body mass index; SD, standard deviation.

^a^ *p*‐value is statistically significant.

Table 2 shows the mean ± SD and ranges for the radiation dosimetry variables: AK and DAP, and the calculated ED based on the ‘UNSCEAR User Manual’ [19]. Group A patients demonstrated a mean ED of 9.5 mSv, while Group B patients, where the protocol was optimised to omit DSA for internal iliac artery visualisation, had a mean ED of 8.7 mSv. The paired sample t‐test for AK showed a statistically significant difference between the groups (*p* < 0.01). Field size (cm^2^) of the detector was also calculated for Group A and B, where the mean ± SD (median) and [range] was found to be 326 ± 631 (216) [142–4460] and 450 ± 978 (205) [120–4886], respectively. The paired sample *t*‐test for field size had a *p*‐value of 0.68 and Cohen's *d* of −0.10.

**TABLE 2:** Radiation dosimetry and conversion of DAP to ED using UNSCEAR user manual [19].

Variable	Group A, mean ± SD (median) [range]	Group B, mean ± SD (median) [range]	Paired sample *t*‐test, *p*, Cohen's *d*
AK (Gy)	0.2 ± 0.1 (0.1) [0.03–0.6]	0.1 ± 0.1 (0.1) [0.01–0.6]	0.006, 0.35^a^
DAP (Gy.cm^2^)	39.7 ± 33 (26.5) [11.7–194]	33.6 ± 39 (23.5) [10.4–261]	0.702, 0.12
ED (mSv)	9.5 ± 6.2 (6.9) [3–31.7]	8.7 ± 10.2 (6.1) [2.7–67.9]	0.698, 0.12

Abbreviations: AK, air kerma; DAP, dose‐area product; ED, effective dose; SD, standard deviation.

^a^ *p*‐value is statistically significant.

**FIGURE 1:**
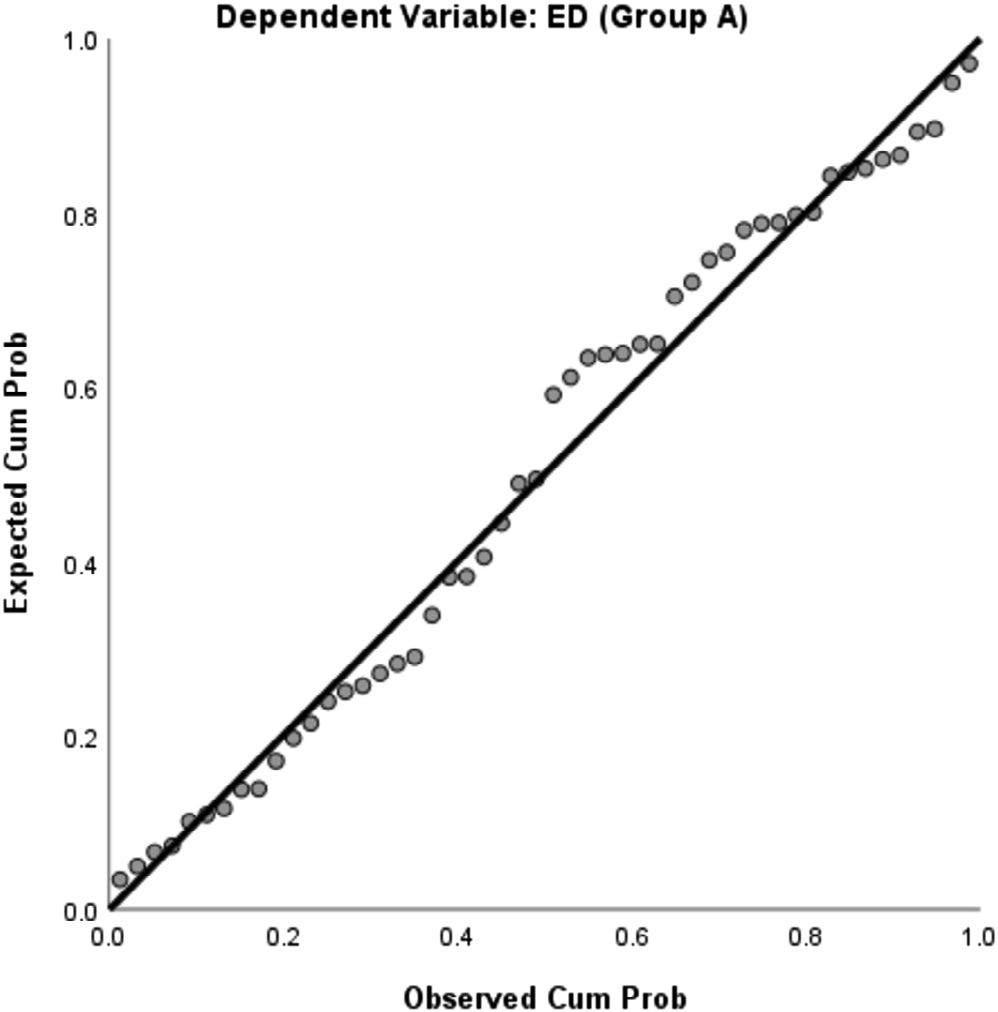
Normal P–P Plot of Regression Standardised Residual for Group A.

**FIGURE 2:**
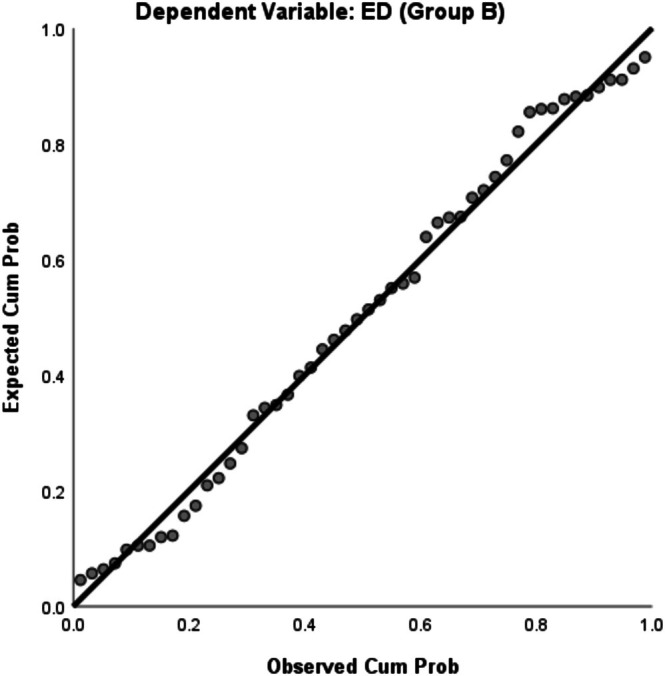
Normal P–P Plot of Regression Standardised Residual for Group B.

Figures 1 and 2 display the normal P–P plots of regression standardised residuals for both Group A and Group B, indicating that the independent variables and dependent variable (i.e., ED) are normally distributed.

Tables 3 and 4 summarise the results of the MVLR analysis for both Groups, showing that DAP was statistically significant (*p* < 0.01).

**TABLE 3:** Multivariable linear regression analysis for Group A patients.

Model	*R* ^2^	Adjusted *R* ^2^ square	SE of estimate	*R* ^2^ change	Change statistics			*p*‐value
					*F* change	df^1^	df^2^	
	1.000	1.000	0.02610	1.000	864018.964	6	43	< 0.001^a^

Model	Unstandardised coefficients					95% confidence interval for *B*		
	*B*	SE	Standardised coefficients beta	*t*	*p*	Lower bound	Upper bound	Collinearity statistics, VIF
(Constant)	0.093	0.048	—	1.924	0.061	−0.005	0.191	—
DAP	0.261	0.000	1.003	568.625	< 0.001^a^	0.260	0.261	16.117
AK	−0.108	0.106	−0.001	−1.022	0.312	−0.322	0.105	9.062
Field size	3.633 × 10^−5^	0.000	−0.003	−1.731	0.091	0.000	0.000	12.617
Age	−0.001	0.001	−0.001	−1.415	0.164	−0.003	0.001	1.135
BMI	−0.001	0.001	−0.000	−0.694	0.491	−0.004	0.002	2.036
Total uterus volume	−4.123 × 10^−5^	0.000	−0.001	−2.430	0.019	0.000	0.000	1.250

*Note:* df^1^, degrees of freedom of numerator; df^2^, degrees of freedom of denominator.

Abbreviations: AK, air kerma; BMI, body mass index; DAP, dose‐area product.

^a^ *p*‐value is statistically significant.

**TABLE 4:** Multivariable linear regression analysis for Group B patients.

Model	*R* ^2^	Adjusted *R* ^2^	SE of estimate	*R* ^2^ change	Change statistics			
					*F* change	df^1^	df^2^	*p*
	1.000	1.000	0.02915	1.000	1001055.939	6	43	< 0.001^a^

Model	Unstandardised coefficients					95% confidence interval for *B*		
	*B*	SE	Standardised coefficients beta	*t*	*p*	Lower bound	Upper bound	Collinearity statistics VIF
(Constant)	−0.032	0.056	—	−0.585	0.561	−0.144	0.079	—
DAP	0.260	0.000	0.999	1505.095	< 0.001^a^	0.260	0.260	2.651
AK	0.027	0.065	0.000	0.415	0.680	−0.105	0.159	2.312
Field size	7.046 × 10^−6^	0.000	0.001	1.063	0.294	0.000	0.000	2.421
Age	0.000	0.001	0.000	−0.324	0.748	−0.002	0.001	1.136
BMI	0.001	0.002	0.000	0.794	0.432	−0.002	0.005	1.649
Total uterus volume	1.15 × 10^−5^	0.000	0.001	1.296	0.101	0.000	0.000	1.258

Abbreviations: AK, air kerma; BMI, body mass index; DAP, dose‐area product.

^a^ *p*‐value is statistically significant.

## 4 Discussion

The multivariable linear regression analysis demonstrated a strong correlation between the reported DAP and the converted ED values in both Group A and Group B. Using the ‘UNSCEAR User Manual’, the DAP‐to‐ED conversion factors served as a valuable estimation tool for determining patient radiation dose exposure with an indication of the effective dose. This method offers a better assessment of radiation dose exposure compared to relying solely on the DAP or AK readings displayed on angiography units, which are widely acknowledged as poor indicators of dose [28]. Effective dose provides a more comprehensive understanding of radiation exposure, enabling improved radiation dose audits in angiography suites where higher doses are typically used compared to other imaging modalities, such as general X‐ray or CT. The ‘UNSCEAR User Manual’ offers a straightforward approach to ED estimation in the clinical setting using the look‐up table (‘Factors for estimation of effective dose from image‐guided interventional procedures’) and calculating the ED (in mSv) with the DAP reading (in the correct units of Gy.cm^2^). The mean ED values for Group A and B were 9.5 and 8.7 mSv, respectively, which are comparable to the radiation dose from a single abdominal CT scan [29]. Although BMI showed a statistically significant paired sample t‐test, it was not a predictor for ED in the MVLR analysis. The BMI ranges in this study cohort correlate with the findings of Brown et al. [30] where the BMI range for Australian women are between 18.5 and 25 kg/m^2^.

Complex IR procedures often involve the use of higher dose image acquisition techniques, including DSA and extensive fluoroscopy to guide patient treatments [9]. Knowledge of radiation dose within the angiography suite remains limited, unless rigorous research is conducted in clinical settings [31]. One study [32] measured the occupational radiation doses during 99 IR procedures and found that prostate artery embolisation procedures exposed interventional radiologists to high radiation doses (median = 15 μSv), with a median staff dose of 3.2 μGy for all procedures One of the most comprehensive studies on radiation doses delivered during IR and neurointerventional procedures was conducted by Miller and colleagues through the Radiation Dose in Interventional Radiology (RAD‐IR) project in 2003 [3, 33]. In Part I of the RAD‐IR project, these researchers [3] measured fluoroscopy time, DAP and cumulative dose (CD; also referred to as AK) in 2142 patients across 35 different types of fluoroscopically guided interventions. At the time, CD was considered the most accurate method for assessing radiation dose. The study reported that CD exceeded 1 Gy for 52% of patients (1108/2142),2 Gy for 30%, 3 Gy for 19%, and 5 Gy for 6% of patients [3]. Given the association of embolisation procedures, transjugular intrahepatic portosystemic shunt, and renal/visceral artery stent placements with clinically significant patient doses, Part II of the RAD‐IR focussed on investigating peak skin dose (PSD) in a subset of these procedures performed on angiography units equipped with skin dose mapping computer software. In 14 UAE patients, PSD was greater than 2 Gy in 50% (*n* = 7). The study concluded that CD data correlated well with the PSD data, making CD a viable substitute metric for PSD in assessing radiation dose exposure [33].

The studies mentioned have used various methods to measure radiation dose exposure, with a particular focus on assessing deterministic effects [3, 32, 33]. Although the International Commission of Radiation Protection does not recommend using ED for risk calculations of individual patients, ED provides valuable insights into population‐level stochastic risks and the evaluation of risks from occupational exposure [28]. Effective dose has been reported for UAE procedures using Monte Carlo simulations. Vetter and colleagues [34] investigated the dosimetry for 70 UAE patients, where radiation dose optimisation was applied to half of the cohort by omitting oblique projections and DSA, using last‐image‐hold (LIH) techniques for roadmapping. The study found that the average DCC for DSA procedures was 0.572, with a mean ED of 29.6 mSv (median 17.1 mSv). For LIH procedures, the average DCC was 0.813, with a mean ED of 10.6 mSv (median 8.1 mSv) [34]. Stratakis et al. [35] estimated organ doses and ED for percutaneous transhepatic biliary procedures in 51 patients, using a Monte Carlo transport code and an adult mathematical phantom. The study reported that the average ED ranged from 1.8 to 5.4 mSv depending on procedural approach and complexity. Additionally, a DAP exposure of 6072 cGy.cm^2^ with 28.3 min of fluoroscopy time corresponded to an ED of 13 mSv [35]. These authors emphasised that operators (i.e., interventional radiologists and radiographers) can make use of DAP‐normalised dosimetric data to calculate ED with a single conversion coefficient, aligning with the design and findings of our study [35]. The ED measurements for both Group A and B in this study have been optimised to minimise the risks of radiation for female patients where radiosensitive organs are exposed to ionising radiation and follow safe and appropriate imaging protocols as outlined by Goodman and Amurao [36].

In the Australian context, studies have used DAP and AK to provide indications of stochastic risks and deterministic effects, respectively, during IR procedures and radiation dose exposure [10, 12]. The ARPANSA has been developing a NDRL through an image‐guided interventional procedures (IGIP) survey conducted across multiple sites nationwide [37, 38]. Common interventional and diagnostic angiography or fluoroscopic procedures in Australia include diagnostic cerebral angiograms, barium and Gastrograffin swallows, EVAR, pelvic embolisations and tunnel line insertions [37]. Similar to NDRLs established in other countries, such as the UK and United States, Australian NDRLs display DAP as the primary diagnostic reference level, allowing sites to compare their dose audit levels [17, 39]. This highlights the need to educate interventional radiologists and radiographers at the frontline of angiographic imaging and the interventional work on utilising resources such as the ‘UNSCEAR User Manual’ and its applicability in clinical settings for simple, non‐complex DAP to ED conversions. This study demonstrated that the relationship between DAP and ED, calculated using the DCCE is strongly correlated, enabling ED calculations to become a part of routine clinical practice and improve clinical radiation dose awareness. The methodology used in this study should be calculated and compared with other IR procedures. Additionally, a revision of the *‘UNSCEAR User Manual’* is also recommended, as the version used in this study, published in October 2017, is now more than 7 years old. Further optimisation of radiation dose is also possible through software and hardware upgrades in angiography suites, as evidenced in the literature comparing different generations of units [12].

The limitations of this study include the absence of a software programme to calculate the effective dose for this IR procedure using known variables from the sample patient data. A suitable Monte Carlo simulation could potentially provide a more accurate measurement of ED, however, the patient data lacked critical geometric information, such as tube angulation, tube voltages (kVp), and source‐to‐image distance (SID) for each projection, which are required for precise modelling. Additionally, this study was limited to a single type of IR procedure performed on a specific body region (pelvis) and a single sex (female), which may not represent the volume or variety of procedures typically encountered in an IR department. Future research could explore a large sample size and broader range of IR procedures performed in angiography suites, encompassing different body regions and patient populations. This data acquisition of Picture Archiving and Communication Systems information on angiography metrics would require use of Python script and coding to run through a Monte Carlo simulation. Moreover, validating the correlation between the ED values obtained from the ‘UNSCEAR User Manual’ and those derived using Monte Carlo simulations would enhance the robustness and applicability of the findings. Finally, the derivatives of the effective dose data and sample demographics included in the ‘UNSCEAR User Manual’ are not well defined, and thus determining site‐specific effective dose calculations is the next logical step for this research.

## 5 Conclusion

This study aimed to evaluate the clinical applicability of DAP‐to‐ED conversion factors to provide interventional radiographers and radiologists an estimation of effective dose for a certain procedure where the patient cohort are mainly reproductive‐aged female patients. Using the ‘UNSCEAR User Manual’, the MVLR analysis demonstrated that the DAP values correlated with the converted ED values for this cohort of patients undergoing uterine artery embolisation procedures. The mean ED (8.7 and 9.5 mSv) was equivalent to the radiation dose of approximately one abdominal CT scan.

Future research into the use of Monte Carlo simulations for various interventional radiology procedures with a collaborative approach between interventional radiographers, medical physicists, and computer scientists is recommended to establish site‐specific DAP‐to‐ED conversion factors to determine effective dose and patient risk to stochastic effects of radiation dose. This can benefit both staff and patients by improving understanding of radiation risk, supporting informed decision‐making and guiding safer clinical practice.

## Ethics Statement

This study was exempted from ethical review by the Adventist HealthCare Limited Human Research Ethics Committee (HREC 2018‐004) as it met the requirements from the National Statement on Ethical Conduct in Human Research 2023; Sections 5.1.17(a).

## Conflicts of Interest

The authors declare no conflicts of interest.

## Data Availability

Data sharing is applicable to the datasets that were analysed during this study.
